# Is the 1-Minute Sit-To-Stand Test a Good Tool to Evaluate Exertional Oxygen Desaturation in Chronic Obstructive Pulmonary Disease?

**DOI:** 10.3390/diagnostics11020159

**Published:** 2021-01-22

**Authors:** Ana L. Fernandes, Inês Neves, Graciete Luís, Zita Camilo, Bruno Cabrita, Sara Dias, Jorge Ferreira, Paula Simão

**Affiliations:** Pulmonology Department, Pedro Hispano Hospital, 4464-513 Matosinhos, Portugal; inesmaria.neves@ulsm.min-saude.pt (I.N.); graciete.teixeira@ulsm.min-saude.pt (G.L.); zita.camilo@ulsm.min-saude.pt (Z.C.); bruno.cabrita@ulsm.min-saude.pt (B.C.); sara.pimentadias@ulsm.min-saude.pt (S.D.); jorge.ferreira@ulsm.min-saude.pt (J.F.); paula.simao@ulsm.min-saude.pt (P.S.)

**Keywords:** COPD, 6MWT, STST, exercise capacity, oxygen desaturation, prognosis

## Abstract

Background: Chronic obstructive pulmonary disease (COPD) is frequently associated with exertional oxygen desaturation, which may be evaluated using the 6-minute walking test (6MWT). However, it is a time-consuming test. The 1-minute sit-to-stand test (1STST) is a simpler test, already used to evaluate the functional status. The aim of this study was to compare the 1STST to the 6MWT in the evaluation of exertional desaturation. Methods: This was a cross-sectional study including 30 stable COPD patients who performed the 6MWT and 1STST on the same day. Six-minute walking distance (6MWD), number of 1STST repetitions (1STSTr), and cardiorespiratory parameters were recorded. Results: A significant correlation was found between the 6MWD and the number of 1STSTr (r = 0.54; *p* = 0.002). The minimum oxygen saturation (SpO_2_) in both tests showed a good agreement (intraclass correlation coefficient (ICC) 0.81) and correlated strongly (r = 0.84; *p* < 0.001). Regarding oxygen desaturation, the total agreement between the tests was 73.3% with a fair Cohen’s kappa (κ = 0.38; *p* = 0.018), and 93.33% of observations were within the limits of agreement for both tests in the Bland–Altman analysis. Conclusion: The 1STST seems to be a capable tool of detecting exercise-induced oxygen desaturation in COPD. Because it is a less time- and resources-consuming test, it may be applied during the outpatient clinic consultation to regularly evaluate the exercise capacity and exertional desaturation in COPD.

## 1. Introduction

Chronic obstructive pulmonary disease (COPD) is a leading cause of morbidity and mortality worldwide, with an economic and social burden that is both substantial and increasing [1]. Sedentarism in COPD is a consequence of constitutional and respiratory symptoms, which progressively affect functional status, exercise capacity, and health-related quality of life. Physical activity limitations and desaturation with exertion are important clinical markers in COPD patients and are associated with a poorer prognosis and higher number of exacerbations [2,3]. 

The 6-minute walking test (6MWT) is considered a validated and reliable test to evaluate the cardiopulmonary and musculoskeletal function in COPD [4]. However, it is not often assessed in investigational, inpatient, outpatient, or primary care settings due to the time, resources, and space needed to conduct it. 

In recent years, new tests have been developed to facilitate exercise capacity in COPD. In this context, the sit-to-stand test (STST) has been proposed. The STST, first described in 1985 by Csuka and McCarty [5], measures a movement commonly performed in everyday life. Two main types of STST have been developed; one measures the number of repetitions in a set time (the 1-minute STST (1STST)) and the other the time taken to perform a set number of repetitions (the 5-repetition STST) [6]. The best protocol, however, was determined to be the 1STST in subjects with COPD [7,8].

The 1STST is accepted as an evaluation tool for the functional status and fall risk prediction of the elderly [9]. In particular, in recent studies, 1STST has shown predictive validity as a strong and independent predictor of mortality and health-related quality of life in COPD patients [10]. Some studies have demonstrated a significant correlation between the number of repetitions (1STSTr) and the 6-min walked distance (6MWD), the quadriceps muscle strength, and the level of physical activity in COPD patients, indicating that the 1STST may be valid for measuring exercise capability after respiratory rehabilitation [11,12,13,14,15,16]. Recently, Crook et al. reported that the 1STST is a reliable test for exercise capacity measurement in COPD with a minimal important difference of three repetitions [13]. Moreover, the 1STST has also been studied to evaluate exercise capacity and oxygen desaturation in interstitial lung disease and cystic fibrosis [17,18].

To our knowledge, a few studies have focused on evaluating the oxygen desaturation during the 1STST in COPD. Therefore, we aimed to compare the 1STST to the 6MWT for the ability to assess exercise-induced oxygen desaturation in COPD.

## 2. Materials and Methods

### 2.1. Subjects

Consecutive patients with COPD, according to the Global Initiative for Chronic Obstructive Lung Disease (GOLD) [1], followed in a pulmonology outpatient department were referred for consideration to our pulmonary function laboratory between August 2017 and February 2019. The exclusion criteria were chronic respiratory failure with long-term oxygen therapy, recent exacerbation (less than one month), patients without a recent lung function test (less than six months), and cardiovascular or orthopedic conditions that limit the ability to perform the tests.

### 2.2. Study Design

The study design was a cross-sectional study including stable COPD patients who performed the 6MWT and 1STST on the same day, with a 30-min resting period and accompanied by a cardiopulmonary technician. The sequence of the tests was random. 

A 6-minute walking distance (6MWD) and the number of repetitions (1STSTr) were assessed. Regarding cardiorespiratory parameters, systolic blood pressure (sBP), diastolic blood pressure (dBP), dyspnea, and lower limb fatigue (modified Borg scale [19]) were recorded before and after both tests. Heart rate (HR) and peripheral arterial saturation (SpO_2_) were continuously measured during the tests and for three minutes after the end of each test using Spirodoc^®^ (Medical International Research, New Berlin, WI, USA). Oxygen desaturation (∆SpO_2_) for each test was defined as the difference between baseline SpO_2_ and the minimum SpO_2_. After a literature review, a ∆SpO_2_ ≥ 4% was considered clinically significant for this study [17].

Demographic, clinical, and lung function data were collected by chart review. Forced vital capacity (FVC), expiratory volume in the first second (FEV_1_), residual volume (RV) and total lung capacity (TLC) were performed by spirometry and plethysmography using Jaeger cabin®. Diffusing capacity for carbon monoxide (DLCO) was measured using the single breath method, and arterial partial pressure of oxygen (PaO_2_) and arterial partial pressure of carbon dioxide (PaCO_2_) from arterial blood gas were obtained.

The protocol of this study was approved by the local ethics committee. The aim of the study was explained to all participants and all signed an informed consent.

### 2.3. Outcome Measurements

#### 2.3.1. Six-Minute Walking Test (6MWT)

Patients performed the 6MWT, according to the guidelines of the European Respiratory Society (ERS)/American Thoracic Society (ATS) [4]. The 6MWT was performed in a 30-meter indoor corridor. A cardiopulmonary technician timed the walk and recorded the distance, using standardized encouragement strategy. None of the patients used a walking aid in daily life. 

#### 2.3.2. One-Minute Sit-to-Stand Test (1STST)

The 1STST was performed in a standard height chair (46 cm) without arm rests, according to the previously described protocols [13]. The test was first demonstrated by the staff and then performed by the participant. The patient was seated upright in the chair with knees and hips flexed at 90°, feet placed flat on the floor at hip width apart, and arms held stationary by placing their hands on their hips. Patients were asked to perform repetitions of standing upright and then sitting down in the same position at a self-paced speed (safe and comfortable) as many times as possible for 1 min. They were instructed not to use their arms for support while rising or sitting. The number of completed repetitions was manually recorded.

### 2.4. Statistical Analysis

All analyses were conducted using SPSS (version 22.0, software IBM, Armonk, NY, USA) for Windows. Descriptive data on continuous variables were reported as mean (M) and standard deviation (SD), depending on the normality of the variable distribution. Normality was verified by asymmetry coefficient and graphic analysis. For categorical variables, absolute (*n*) and relative (%) frequencies were used. The relationship between the 6MWD and the 1STSTr and the minimum SpO_2_ of both tests was calculated by Pearson’s correlation coefficient. This coefficient was also used to evaluate a relationship between 6MWD, 1STSTr, or minimum SpO_2_ of both tests and clinical or lung function variables. The correlation value (r) was evaluated as: 0–0.25 = very weak, 0.26–0.50 = weak, 0.51–0.75 = moderate, 0.76–0.90 = strong, and 0.91–1.0 = very strong. The ANOVA test was used to measure the differences and evolution in the cardiorespiratory parameters, dyspnea, and lower limb fatigue (modified Borg scale) during the 6MWT and the 1STST. Agreement between the values obtained in the 6MWT and the 1STST were evaluated with the intraclass correlation coefficient (ICC). Agreement between the ability of the two exercise tests to detect desaturation ≥4% was assessed using Cohen’s kappa coefficient (κ). The κ values of < 0, 0–0.20, 0.21–0.40, 0.41–0.60, 0.61–0.80, and 0.81–1 were considered to indicate no, slight, fair, moderate, substantial, and almost perfect agreement, respectively. A Bland–Altman analysis was conducted to graphically represent the limits of agreement between the minimum SpO_2_ in the 6MWT and 1STST. A *p* value of less than 0.05 was considered significant. 

## 3. Results

### 3.1. Participants

Thirty patients were included in the analysis, mainly males (26–86.7%) with a mean age of 67.57 ± 9.10 years and former smokers (15–50.0%). The mean body mass index (BMI) was 25.17 ± 4.98 kg/m^2^. Most patients were included in the moderate-to-severe GOLD category [1] (GOLD 1: 1 (3.3%), GOLD 2: 16 (53.3%), GOLD 3: 12 (40%), GOLD 4: 1 (3.3%)). Demographic, clinical, and lung function characteristics are represented in Table 1.

### 3.2. Comparison of Exercise Capacity and Cardiorespiratory Parameters between the 1STST and the 6MWT

The average number for the 1STSTr was 18.13 ± 5.46 in our population (Table 1). Regarding the 1STST, the number of repetitions presented a negative and statistical correlation with age (r = −0.53; *p* = 0.002), but it did not correlate with BMI or lung function parameters (FVC, FEV1, RV, TLC, or DLCO).

The mean 6MWD was 409.37 ± 103.23 m (Table 1). There was a statically negative correlation between 6MWD with age (r = −0.36; *p* = 0.049) and a positive correlation with FVC (r = 0.37; *p* = 0.044) and DLCO (r = 0.40; *p* = 0.036). No correlation was found with BMI or other lung function parameters.

**Figure 1:**
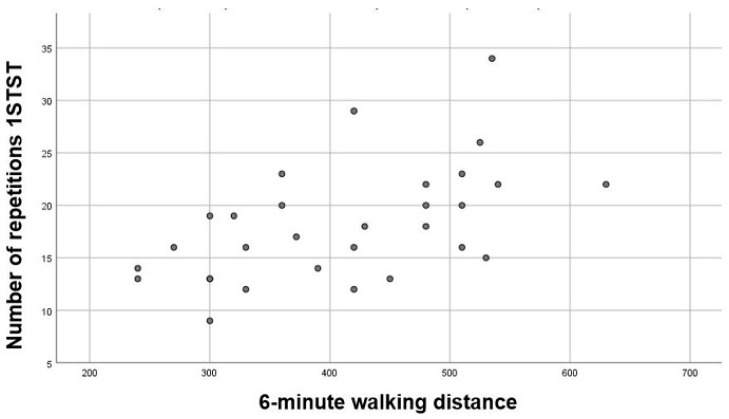
Correlation between 6MWD and 1STSTr (1STSTr = number of repetitions during 1-min sit-to-stand test; 6MWD = 6-min walking distance).

Furthermore, there was a statistically significant positive correlation between the 6MWD and the 1STSTr (r = 0.54; *p* = 0.002) (Figure 1).

**Figure 2:**
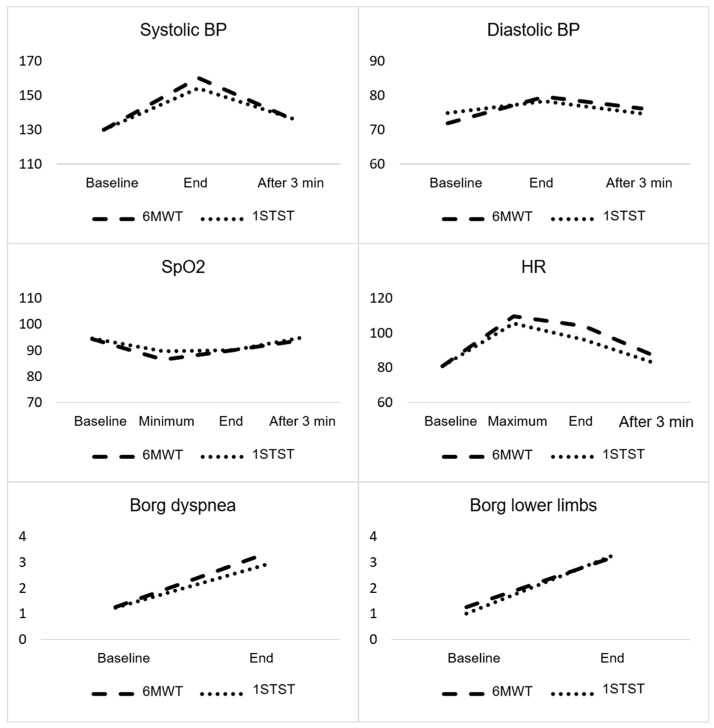
Evolution of cardiorespiratory parameters, Borg dyspnea, and lower limb fatigue during the 6MWT and the 1STST. (1STST = 1-min sit-to-stand test; 6MWT = 6-min walking test; sBP = systolic blood pressure; dBP = diastolic blood pressure; HR = heart rate; SpO_2_ = oxygen saturation).

The results of the cardiorespiratory parameters, Borg dyspnea, and lower limb fatigue obtained during the 1STST and 6MWT are presented in Table 2. The evolution of the parameters resembles a polynomial function of the quadratic type (Figure 2). A significant increase in systolic blood pressure (sBP) (*p* < 0.001), diastolic blood pressure (dBP) (*p* < 0.001), heart rate (HR) (*p* < 0.001), Borg dyspnea (*p* < 0.001), and Borg lower limb fatigue (*p* < 0.001) were noted during the 6MWT and the 1STST. A significant decrease in SpO_2_ was observed with both tests, as well as an increase in HR. After three minutes, the previously stated variables were similar to the baseline values. An interaction effect was observed in the evolution of sBP that registered a higher value at the end of the 6MWT (M = 160.17; SD = 21.93) compared to the 1STST (M = 154.17; SD = 23.77). The evolution of dBP (*p* = 0.182), HR (*p* = 0.126), SpO_2_ (*p* = 0.148), Borg dyspnea (*p* = 0.103), and Borg lower limbs’ fatigue (*p* = 0.238) was similar in both groups.

When analyzing the differences between the 6MWT and the 1STST, we verified that the HR obtained a slightly higher but statistically significant value in the 6MWT (*p* = 0.007). Regarding SpO_2_, the 6MWT obtained a significantly lower value comparing to the 1STST (*p* = 0.02).

The agreement values for cardiorespiratory parameters between the 6MWT and the 1STST are presented in Table 3. Moderate or strong agreement values were obtained for most variables. The minimum SpO_2_ showed a substantial agreement between tests (ICC = 0.810) as did the end-test Borg dyspnea (ICC 0.750).

### 3.3. Comparison of Oxygen Desaturation between the 1STST and 6MWT 

The minimum SpO_2_ on STST was 89.73 ± 5.01 and 18 patients (60.0%) presented an oxygen desaturation (∆SpO_2_) ≥ 4%. Four patients (13.3%) registered their minimum SpO_2_ during the three minutes after the end of the test, mostly during the first minute. There were statistical correlations between minimum SpO_2_ in 1STST with TLC (r = 0.41; *p* = 0.025) and paO2 (r = 0.52; *p* = 0.007). No correlation was found with age, BMI, or other lung function parameters.

The minimum SpO_2_ on 6MWT was 86.47 ± 6.55 and 26 patients (86.7%) presented a desaturation (∆SpO_2_) ≥ 4%. In one participant (3.0%), the minimum SpO_2_ was obtained after the end of the test. A statistically significant correlation was verified between minimum SpO_2_ in the 6MWT with paO2 (r = 0.67; *p* < 0.001), FVC (r = 0.38; *p* = 0.036), FEV1 (r = 0.38; *p* = 0.037), and DLCO (r = 0.38; *p* = 0.043). No correlation was found with age, BMI, and other lung function parameters. Individual SpO_2_ measurements during the 6MWT and the 1STST are presented in Table S1.

**Figure 3:**
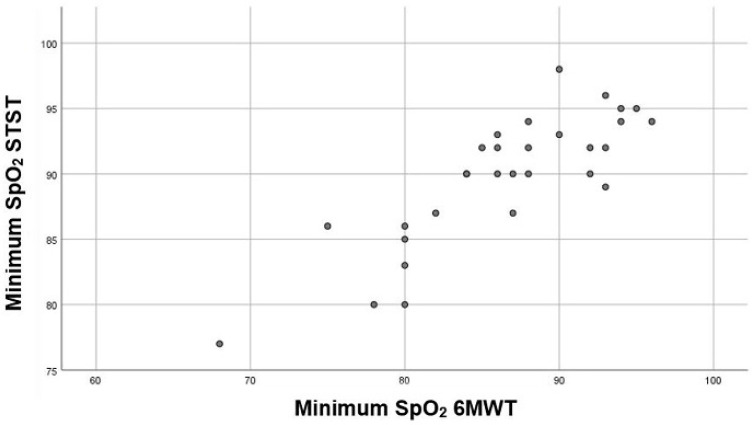
Correlation between minimum SpO_2_ registered during the 1STST and the 6MWT. (SpO_2_ = oxygen saturation; 1STST = 1-min sit-to-stand test; 6MWT = 6-min walking test).

A significant, strong, and positive correlation was found between the minimum SpO_2_ registered in the 1STST and the 6MWT (r = 0.84; *p* < 0.001) (Figure 3).

Regarding oxygen desaturation (∆SpO_2_), the total agreement between the 1STST and the 6MWT was 73.3% with a fair Cohen’s kappa, as shown in Table 4.

**Figure 4:**
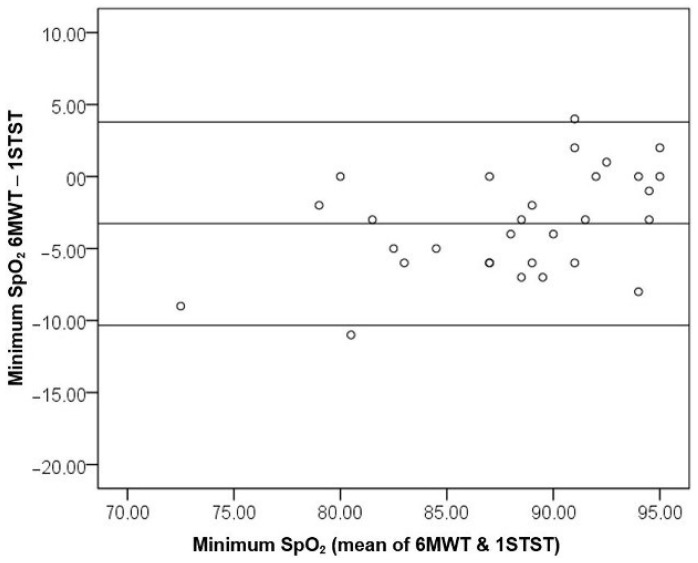
Bland–Altman plot of the difference in the minimum SpO_2_ between the 6MWT and 1STST as a function of the mean minimum SpO_2_ in both tests. (SpO_2_ = oxygen saturation; 6MWT = 6-min walking test; 1STST = 1-min sit-to-stand test).

Moreover, the agreement between the minimum SpO_2_ values of the two tests was evaluated by the Bland–Altman plot (Figure 4). The limit of agreement was calculated as M ± 1.96×SD, which created the interval ] −3.27 − 1.96 × 3.60; −3.27 + 1.96 × 3.60 [=] −10.33; 3.79 × [. A total of 93.33% of observations were within the limits of agreement, as demonstrated in Figure 4.

## 4. Discussion

The purpose of this study was to compare the utility of the 1STST with the 6MWT in the evaluation of exertional oxygen desaturation in patients with COPD. Our study established that the 1STST seems to be a reliable test to estimate exercise-induced oxygen desaturation in COPD, as demonstrated by the existence of a strong correlation and agreement between minimum SpO_2_ in the 1STST and in the 6MWT and a good total concordance between oxygen desaturation recorded during both tests.

In various chronic respiratory disorders, mortality and morbidity have been associated with measurements during the 6MWT or the cardiopulmonary exercise testing (CPET), including oxygen desaturation and minimum SpO_2_. This suggests that exercise-induced oxygen desaturation may be a key element in the prognostic evaluation and follow-up in COPD [20,21,22]. Moreover, some studies have demonstrated that regular monitoring of physiological variables during effort, such as oxygen desaturation and heart rate, may help to detect an exacerbation earlier [3,23].

The 6MWT is acknowledged as a simple test to evaluate exercise capacity and de-saturation on exertion. As COPD is a highly prevalent disease [1], the follow-up of many patients is widely performed by the general practitioner or in office-based practices. In these settings, it may be difficult to guarantee the necessary conditions to complete a 6MWT, which may contribute to the undervaluation of exercise capacity.

The 1STST is less time consuming and does not need specific equipment, as it may be performed at the outpatient setting using a portable oximeter. Our results demonstrate that moderate-to-severe COPD patients (GOLD 2 and 3) performed a mean of 18.13 ± 5.46 repetitions. Moreover, a moderate, positive, and significant correlation was found between 6MWD and the 1STSTr. This is in line with previous publications and corroborates the utility of the 1STST for evaluating exercise capacity [11,12,13].

To our knowledge, few studies have focused on evaluating oxygen desaturation with the 1STST in COPD. Contradicting results regarding the SpO_2_ decline in 1STST have been described in the literature. On the one hand, studies by Meriem and Ozalevli did not report a significant change of SpO_2_ during the STST in their publications [11,12]. On the other hand, Crook et al. observed that the SpO_2_ during the 1STST may continue to decline after the end of the test. When using the minimum SpO_2_ instead of the end-test SpO_2_, they detected desaturation during the 1STST in all patients who had desaturation in the 6MWT [13]. In our study, we verified a significant decrease in SpO_2_ during the 6MWT and the 1STST; however, the oxygen desaturation was higher during the 6MWT, which is similar to previous articles [13,17,18]. This could be due to the shorter duration of the 1STST or the higher muscle demand during the 6MWT. We also demonstrated that the minimum SpO_2_ was concordant in both tests and strongly correlated. Furthermore, we observed that the 1STST and the 6MWT could detect a desaturation ≥4%, which was considered a significant threshold for SpO_2_ variability in both tests.

In some studies, the cardiovascular demand seemed to be greater during the 6MWT than in the 1STST [11,12]. Other publications have demonstrated a comparable end-exercise HR response in both tests, but blood pressure parameters were not evaluated [13,17]. We reported a higher increase in sBP, dBP, and HR in 6MWT than in the 1STST; however, the results only reached a statistically significant difference for HR. Duration and demand of peripheral muscles is different between these tests, which may partially explain the distinct cardiovascular response [11,12]. Lower limb fatigue was not statistically different in our study, but the mean result obtained was slightly higher at the end of the 1STST.

We tried to explore the results obtained in both tests and the clinical and functional measurements. Age was correlated with impaired exercise capacity in 1STST and 6MWT in concordance with previous studies. No correlation was found between 1STST outcomes and lung function parameters. Despite the fact that FEV1 has been considered a poor predictor of disease status, lung function is routinely used to assess COPD severity. A correlation between lung function and the 1STST would mean that the 1STSTr or the minimum SpO_2_ could be useful severity surrogates as the 1STST is an easier test to perform. 

Several STST protocols have been published in the literature [7]. The availability of multiple protocols has probably slowed the acceptance by the scientific community and the development of standardized criteria for this test. The 1STST is the most used test and seems to be practical enough for regular usage in the daily outpatient routine. Furthermore, it is sufficiently long to detect oxygen desaturation [13,17]. In some patients, the SpO_2_ continues to decline after the end of the test, although it is fundamental to maintain a continuous SpO_2_ monitoring during and for some minutes after the end of the test [13].

The main limitation of our study was the sample size and the absence of a control group of healthy subjects for comparison with the 1STST results. Recently, the 1STST was validated in a COPD cohort for measuring functional capacity, and reference values for the 1STST were published. However, no validity for oxygen desaturation or SpO_2_ values were studied [13,24]. Another limitation was the possibility of an impact on results according to the order of the tests. A learning effect of 27 meters has been reported for the 6MWT and of 0.8 repetitions for the 1STST in COPD [13,25]. However, we found no studies evaluating this subject and in our study the order of the tests was completely random to minimize this possible effect on SpO_2_.

In the future, it would be interesting to assess the impact of using 1STST as a triage tool to prioritize a formal evaluation of oxygen desaturation with a 6MWT or CPET in COPD patients. Another attractive possibility may be to perform 1STST sequentially to assess its effect on exacerbations and survival in COPD. Additionally, the role of the 1STST may also be studied in other chronic respiratory and cardiovascular disorders.

## 5. Conclusions

Our study highlights the ability of the 1STST to detect exercise-induced oxygen desaturation. The 1STST is an easy-to-perform and well-tolerated test and does not need specialized equipment (only a portable oximeter). Hence, it is ideal for implementation by general practitioners and outpatient routine consultations to evaluate exercise capacity oxygen desaturation, which is a negative marker for COPD prognosis. Larger studies will be needed to confirm our described results in COPD.

## Figures and Tables

**Table 1:** Patients’ baseline characteristics, lung function tests, and results of the 1STST and the 6MWT. (M = mean; SD = standard deviation; 1STST = 1-min sit-to-stand test; 6MWT = 6-min walking test; mMRC = modified Medical Research Council; BODE = index with BMI, FEV_1_, 6MWD, and mMRC; L= liters).

Variable	M/n	SD/%	Minimum	Maximum
Male gender	26	86.7	--	--
Smoking status Former smoker | smoker | non-smoker	15|13|2	50.0|43.3|6.7	--	--
Age (years)	67.57	9.10	48	83
Body mass index (BMI) (kg/m^2^)	25.17	4.98	15.05	36.05
mMRC dyspnea scale	1.60	0.81	0	3
BODE index	2.70	1.75	0	6
Forced vital capacity (FVC) (liters (L))	2.51	0.76	1.42	4.56
Forced vital capacity (% predicted)	73.05	15.31	48.00	107.00
Forced expiratory volume in 1st second (FEV_1_) (L)	1.41	0.52	0.60	2.78
Forced expiratory volume in 1st second (% predicted)	50.83	14.49	24.70	78.00
Residual volume (RV) (L)	4.47	1.43	2.66	9.31
Residual volume (% predicted)	191.87	58.32	106.00	354.00
Total lung capacity (TLC) (L)	7.09	1.77	4.87	12.59
Total lung capacity (% predicted)	116.37	19.97	78.90	172.00
Diffusing capacity for carbon monoxide (DLCO) (% predicted)	45.14	20.47	15.00	100.00
Arterial partial pressure of oxygen (PaO2) (mmHg)	69.80	13.80	34.00	91.00
Arterial partial pressure of carbon dioxide (PaCO2) (mmHg)	44.36	7.47	33.00	64.00
6-minute walking distance (6MWD) (meters)	409.37	103.23	240.00	630.00
Percentage of 6MWD (%) Baseline SpO_2_ 6MWT (%) Minimum SpO_2_ 6MWT (%)	84.23 94.47 86.47	20.65 2.60 6.55	52.00 88.00 68.00	134.00 99.00 96.00
Number of repetitions during 1STST (1STSTr) Baseline SpO_2_ 1STST (%) SpO_2_ minimum 1STST (%)	18.13 94.67 89.73	5.46 2.58 5.01	9.00 89.00 77.00	34.00 99.00 98.00

**Table 2:** ANOVA of repetitive measurements for the evolution of systolic blood pressure, diastolic blood pressure, heart rate, oxygen saturation, Borg dyspnea, and lower limbs’ fatigue in the 6MWT and the 1STST. (1STST = 1-min sit-to-stand test; 6MWT = 6-min walking test; sBP = systolic blood pressure; dBP = diastolic blood pressure; HR = heart rate; SpO_2_ = oxygen saturation).

Variable	Test	Baseline	Maximum Minimum	End	After 3 min	Variance Analysis		
						Evolution	Test	Interaction
sBP ^&^	6MWT	129.93 (13.38)	--	160.17 (21.93)	136.20 (17.72)	*p* < 0.001 *	*p* = 0.179	*p* = 0.005 *
	STST	130.17 (17.38)	--	154.17 (23.77)	136.33 (20.16)			
dBP ^&^	6MWT	71.87 (9.70)	--	79.67 (10.68)	76.27 (10.64)	*p* < 0.001 *	*p* = 0.941	*p* = 0.182
	STST	74.87 (11.80)	--	78.37 (10.68)	74.83 (10.54)			
HR ^&^	6MWT	81.03 (14.24)	109.80 (14.24)	104.03 (16.04)	87.00 (12.66)	*p* < 0.001 *	*p* = 0.007 *	*p* = 0.126
	STST	80.87 (13.69)	105.60 (15.09)	96.13 (14.36)	82.60 (12.60)			
SpO_2_^&^	6MWT	94.47 (2.60)	86.47 (6.55)	90.00 (6.80)	94.03 (2.91)	*p* < 0.001 *	*p* = 0.020 *	*p* = 0.148
	STST	94.67 (2.58)	89.73 (5.01)	90.10 (5.42)	95.03 (2.81)			
Borg Dyspnea ^#^	6MWT	1.27 (0.91)	--	3.33 (2.06)	--	*p* < 0.001 *	*p* = 0.095	*p* = 0.103
	STST	1.23 (0.97)	--	2.90 (1.77)	--			
Borg Limbs ^#^	6MWT	1.27 (0.87)	--	3.23 (1.89)	--	*p* < 0.001 *	*p* = 0.668	*p* = 0.238
	STST	1.00 (0.83)	--	3.30 (1.64)	--			

Values represented as mean and standard deviation; ^&^ quadratic adjustment; ^#^ linear adjustment; * statistically significant.

**Table 3:** Agreement on cardiorespiratory parameters between 1STST and 6MWT evaluated by intraclass correlation coefficient (ICC). (1STST = 1-min sit-to-stand test; 6MWT = 6-min walking test; sBP = systolic blood pressure; dBP = diastolic blood pressure; HR = heart rate; SpO_2_ = oxygen saturation).

Variable	Baseline	Minimum	Maximum	End	After 3 min
sBP	0.780	-	-	0.870	0.866
dBP	0.739	-	-	0.754	0.340
HR	0.891	-	0.665	0.484	0.789
SpO_2_	0.833	0.810	-	0.506	0.764
Borg dyspnea	0.942	-	-	0.750	-
Borg limbs	0.669	-	-	0.555	-

**Table 4:** Agreement between ∆SpO_2_ ≥ 4% in the 1STST and the 6MWT, evaluated by Cohen’s kappa (k). (1STST = 1-min sit-to-stand test; 6MWT = 6-min walking test; SpO_2_ = oxygen saturation).

Variables	∆SpO_2_ ≥ 4% (1STST)		Agreement Analysis
∆SpO_2_ ≥ 4% (6MWT)	No	Yes	% total agreement = 73.3% (*p* = 0.018) Cohen’s kappa = 0.38
No	4 (13.3%)	0 (0%)	
Yes	8 (26.7%)	18 (60.0%)	

## Supplementary Materials

The following are available online at https://www.mdpi.com/2075-4418/11/2/159/s1, Table S1: Individual SpO_2_ measurements during the 6MWT and the 1STST. (SpO_2_: oxygen saturation; 6MWT: 6-minute walking test; 1STST: 1-minute sit-to-stand test).
