# Supplementary material for: Is the 1-Minute Sit-To-Stand Test a Good Tool to Evaluate Exertional Oxygen Desaturation in Chronic Obstructive Pulmonary Disease?
Source: Diagnostics (Basel). 2021 Jan 22;11(2):159. doi: 10.3390/diagnostics11020159 (PMC7911810; doi:10.3390/diagnostics11020159)
Supplement: Supplementary file 1 [file diagnostics-11-00159-s001.pdf]

Supplementary Materials

Table S1. Individual SpO2 measurements during the 6MWT and the 1STST. (SpO_2: oxygen saturation; 6MWT: 6-minute walking test; 1STST: 1-minute sit-to-stand test).

Patient	Baseline SpO_2 6MWT	End SpO_2 6MWT	Mininum SpO_2 6MWT	After 3m SpO2 6MWT	Baseline SpO_2 1STST	End SpO_2 1STST	Mininum SpO_2 1STST	After 3m SpO_2 1STST
1	94	92	90	93	95	93	93	95
2	97	95	93	96	99	74	96	98
3	97	96	96	97	96	96	94	97
4	94	93	86	94	94	94	90	95
5	97	92	84	93	97	94	90	96
6	94	86	80	90	92	91	83	90
7	90	82	80	90	92	87	86	90
8	93	90	85	93	93	87	92	94
9	93	88	82	94	92	87	87	94
10	95	83	80	94	95	80	80	93
11	95	85	84	94	96	90	90	94
12	98	98	92	98	98	90	90	99
13	90	82	80	91	90	88	85	90
14	94	89	87	94	94	92	87	95
15	89	70	68	85	89	83	77	88
16	97	95	93	97	97	92	92	96
17	97	97	93	97	95	89	89	97
18	95	94	87	97	96	90	90	97
19	94	90	86	93	95	96	93	98
20	96	97	95	97	96	95	95	97
21	95	93	86	93	97	92	92	98
22	88	79	75	93	91	86	86	92
23	95	95	92	95	95	92	92	95
24	94	94	88	95	94	92	92	95
25	97	96	94	97	97	96	95	97
26	94	90	88	96	94	90	90	96
27	95	90	88	95	97	94	94	98
28	95	92	90	92	98	98	98	98
29	99	99	94	98	96	95	94	96
30	93	78	78	90	90	80	80	93
